# Supplementary material for: Intragranular cracking as a critical barrier for high-voltage usage of layer-structured cathode for lithium-ion batteries
Source: Nat Commun. 2017 Jan 16;8:14101. doi: 10.1038/ncomms14101 (PMC5241805; doi:10.1038/ncomms14101)
Supplement: Supplementary Information — Supplementary Figures [file ncomms14101-s1.pdf]

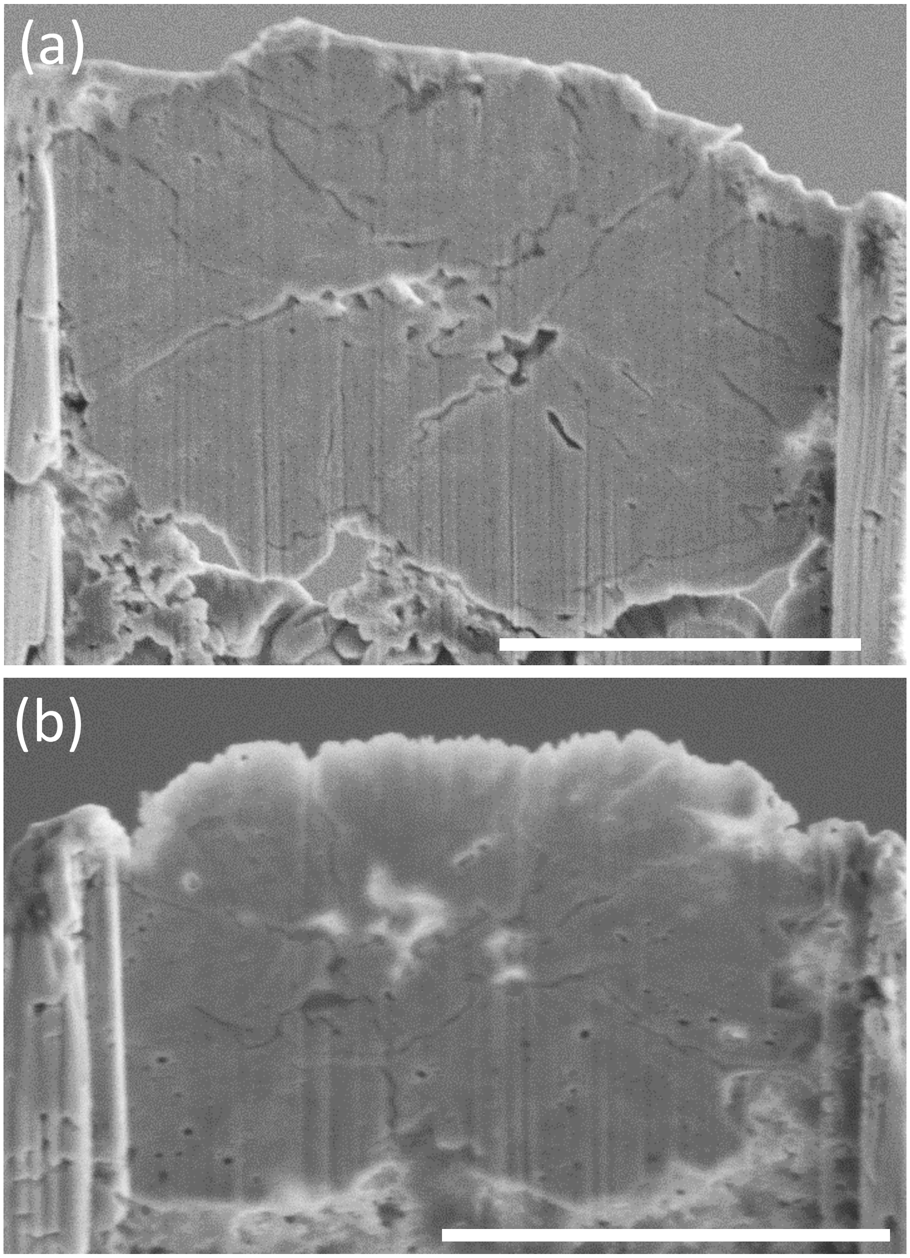

Supplementary Figure 1. Cross sectional scanning electron microscopy (SEM) images. SEM images reveal the Intergranular cracks formation in the $LiNi_{1/3}Mn_{1/3}Co_{1/3}O_2$ (NMC333) secondary particles following the battery cycling at different voltages. (a) after 100 cycles with the high cut-off voltage of 4.2 V and (b) after 100 cycles with the high cut-off voltage of 4.5 V. The scale bars are 5 µm.

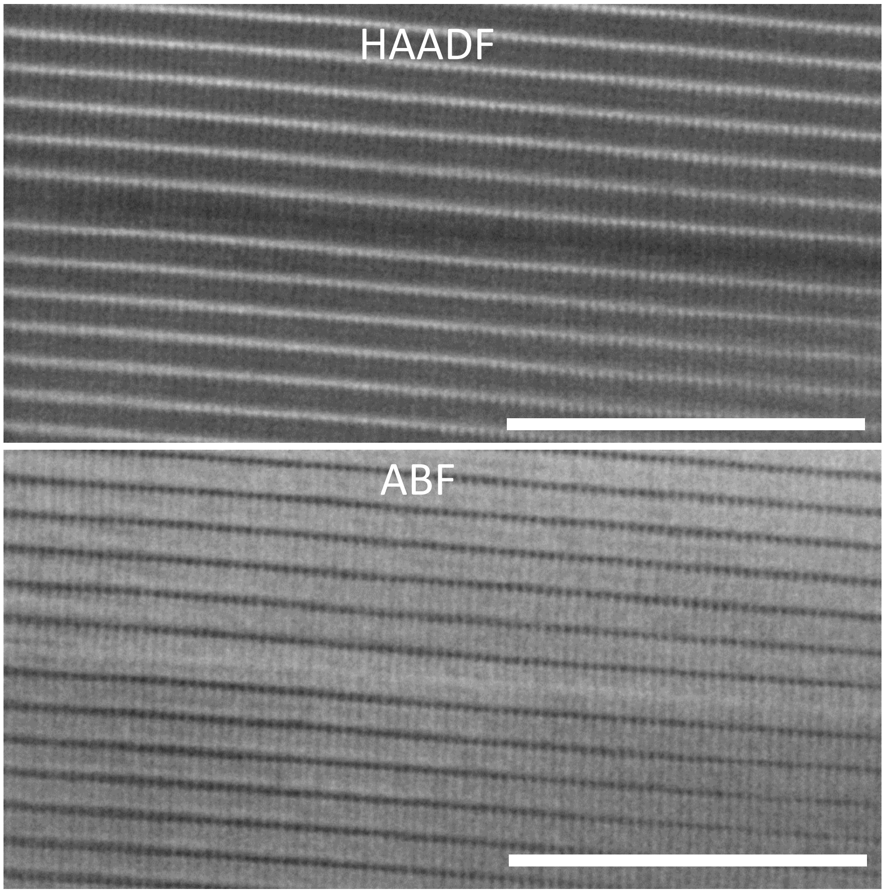

Supplementary Figure 2. Imaging a premature crack. High angle annular dark field/annular bright field (HAADF/ABF) images of a premature crack along the [1-10] zone axis in a $LiNi_{1/3}Mn_{1/3}Co_{1/3}O_2$ (NMC333) particle following 100 cycles with the high cut-off voltage of 4.5 V. The contrast within the strip in the ABF image indicates the strip is not empty. The scale bars are 5 nm.

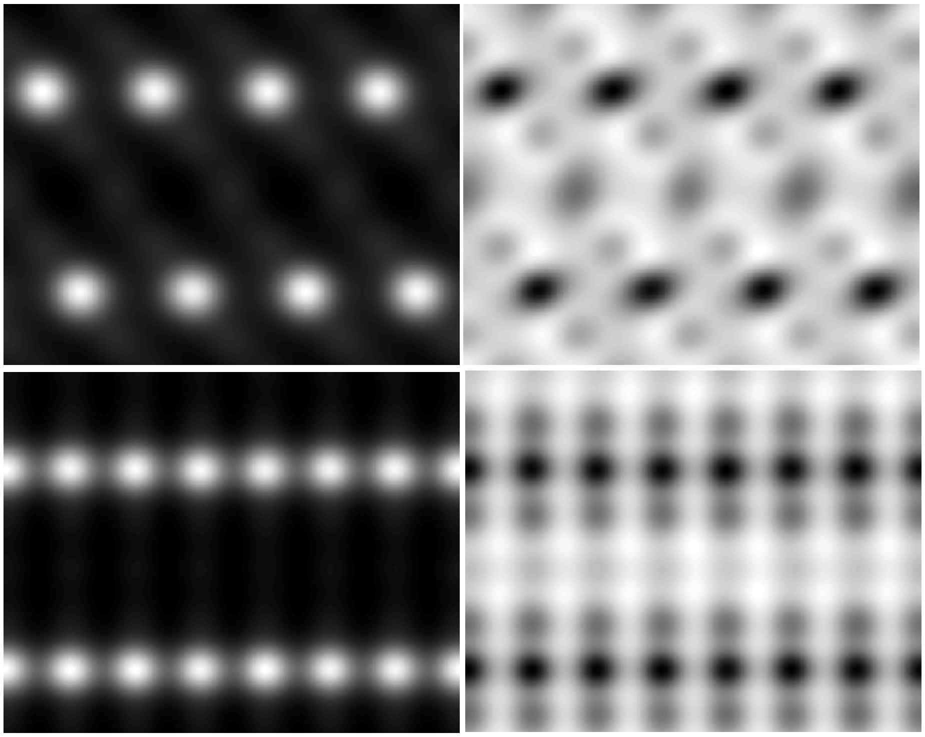

Supplementary Figure 3. Image simulation. Simulated high angle annular dark field/annular bright field (HAADF/ABF) images from [010] zone axis (top row) and [1-10] zone axis (bottom row). The sample thickness is 50 nm.

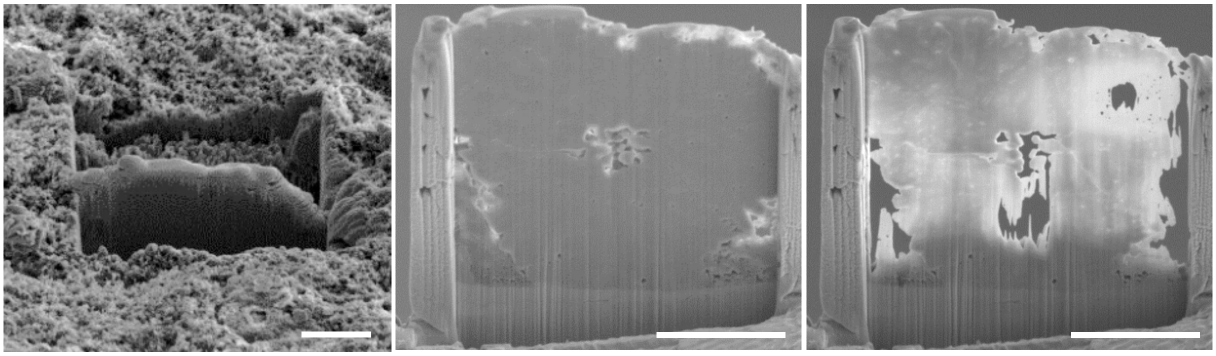

Supplementary Figure 4. Specimen preparation. Scanning electron microscopy images to show the lift-out process in preparation of a transmission electron microscopy (TEM) specimen by using focused ion beam (FIB). The scale bars are 4 nm.
